# Muscarinic Modulation of Morphologically Identified Glycinergic Neurons in the Mouse PreBötzinger Complex

**DOI:** 10.3389/fncel.2019.00562

**Published:** 2020-01-09

**Authors:** Fang Zheng, Barbara E. Nixdorf-Bergweiler, Elke Edelmann, Johannes F. M. van Brederode, Christian Alzheimer

**Affiliations:** ^1^Institute of Physiology and Pathophysiology, Friedrich-Alexander-Universität Erlangen-Nürnberg, Erlangen, Germany; ^2^Institut für Physiologie, Otto-von-Guericke-Universität, Magdeburg, Germany; ^3^Department of Physiology and Biophysics, University of Washington, Seattle, WA, United States

**Keywords:** muscarinic acetylcholine receptors, inhibitory neurotransmission, glycine, preBötzinger complex, morphometric analysis

## Abstract

The cholinergic system plays an essential role in central respiratory control, but the underlying mechanisms remain elusive. We used whole-cell recordings in brainstem slices from juvenile mice expressing enhanced green fluorescent protein (EGFP) under the control of the glycine transporter type 2 (GlyT_2_) promoter, to examine muscarinic modulation of morphologically identified glycinergic neurons in the preBötzinger complex (preBötC), an area critical for central inspiratory rhythm generation. Biocytin-filled reconstruction of glycinergic neurons revealed that the majority of them had few primary dendrites and had axons arborized within their own dendritic field. Few glycinergic neurons had axon collaterals extended towards the premotor/motor areas or ran towards the contralateral preBötC, and had more primary dendrites and more compact dendritic trees. Spontaneously active glycinergic neurons fired regular spikes, or less frequently in a “burst-like” pattern at physiological potassium concentration. Muscarine suppressed firing in the majority of regular spiking neurons *via* M_2_ receptor activation while enhancing the remaining neurons through M_1_ receptors. Interestingly, rhythmic bursting was augmented by muscarine in a small group of glycinergic neurons. In contrast to its heterogeneous modulation of glycinergic neuronal excitability, muscarine generally depressed inhibitory and excitatory synaptic inputs onto both glycinergic and non-glycinergic preBötC neurons, with a stronger effect on inhibitory input. Notably, presynaptic muscarinic attenuation of excitatory synaptic input was dependent on M_1_ receptors in glycinergic neurons and on M_2_ receptors in non-glycinergic neurons. Additional field potential recordings of excitatory synaptic potentials in the M_2_ receptor knockout mice indicate that glycinergic and non-glycinergic neurons contribute equally to the general suppression by muscarine of excitatory activity in preBötC circuits. In conclusion, our data show that preBötC glycinergic neurons are morphologically heterogeneous, and differ in the properties of synaptic transmission and muscarinic modulation in comparison to non-glycinergic neurons. The dominant and cell-type-specific muscarinic inhibition of synaptic neurotransmission and spiking may contribute to central respiratory disturbances in high cholinergic states.

## Introduction

The preBötzinger complex (preBötC) in the ventrolateral medulla contains neurons that generate the inspiratory phase of the respiratory rhythm (Smith et al., 1991; Rekling and Feldman, 1998; Richter and Smith, 2014). The preBötC is composed of glutamatergic excitatory neurons and GABAergic/glycinergic inhibitory neurons (Winter et al., 2009). The crucial role of excitatory glutamatergic neuronal circuits in rhythm generation is well-established (Feldman and Smith, 1989; Greer et al., 1991; Del Negro et al., 2009). A subpopulation of GABAergic and glycinergic neurons in the preBötC can also act as pacemaker neurons (Kuwana et al., 2006; Morgado-Valle et al., 2010), but the overall function of preBötC inhibitory neurons remains to be determined (Abdala et al., 2015; Harris et al., 2017). Local application of strychnine, an antagonist of glycine receptors, into the preBötC, severely disrupts the normal respiratory pattern (Pierrefiche et al., 1998), indicating, together with other studies, that glycinergic inhibition is important for shaping respiratory patterns (Feldman and Smith, 1989; Shao and Feldman, 1997; Janczewski et al., 2013; Shevtsova et al., 2014; Sherman et al., 2015; Fortuna et al., 2019). As a consequence, defects in glycinergic neurotransmission were linked to respiratory pathologies (Büsselberg et al., 2001; Hülsmann et al., 2019).

The frequency, regularity and amplitude of the respiratory cycle are under the control of multiple endogenously released neuromodulators, including acetylcholine (Bellingham and Ireland, 2002; Shao and Feldman, 2005; Zanella et al., 2007; Doi and Ramirez, 2008; Tryba et al., 2008; Koch et al., 2013). Suppression of acetylcholinesterase activity increases the frequency of respiratory rhythmic activity (Shao and Feldman, 2005). Organophosphate pesticide poisoning, which inhibits acetylcholinesterase, has been implicated in respiratory failure (Carey et al., 2013), and neurological disorders such as sudden infant death syndrome and sleep apnea have been linked to impaired muscarinic respiratory control (Kinney et al., 1995; Kubin and Fenik, 2004). Cholinergic receptors are distributed throughout the brainstem, and both muscarinic and nicotinic mechanisms participate in central cholinergic respiratory control (Shao and Feldman, 2005, 2009). Among the five G protein-coupled muscarinic acetylcholine receptors (mAChRs, M_1_R–M_5_R; Wess, 1996; Caulfield and Birdsall, 1998), M_2_Rs dominate in the pons and brainstem (Levey, 1993). M_2_Rs and M_3_Rs are abundant in the preBötC region (Lai et al., 2001), and activation of M_3_Rs leads to depolarization in inspiratory neurons in the rhythmic neonatal rat medullary slice preparation (Shao and Feldman, 2000).

Since a substantial number of inspiratory neurons in the preBötC are glycinergic (Winter et al., 2009, 2010; Koizumi et al., 2013), we focused on the muscarinic modulation of this subpopulation of neurons. We identified these cells by fluorescent labeling with an enhanced green fluorescent protein (EGFP) for the neuronal glycine transporter type 2 (GlyT_2_; Zeilhofer et al., 2005). By combining morphological and electrophysiological techniques in a brainstem slice preparation, we describe the diverse axonal projections of preBötC glycinergic neurons and a predominant M_2_R-mediated attenuation of action potential (AP) discharge. Furthermore, in contrast to the wealth of data on the preBötC involvement in an inspiratory rhythmic generation, little is known about the functional properties of excitatory and inhibitory synapses onto preBötC neurons. Our recordings in the preBötC revealed that glycinergic and non-glycinergic neurons differ in the synaptic properties and that synaptic transmission in both populations of neurons is depressed by mAChR activation. Unexpectedly, we found that mAChRs control excitatory synaptic drive onto glycinergic and non-glycinergic preBötC neurons *via* different receptor subtypes.

## Materials and Methods

### Animals and Brain Slice Preparation

Experiments were performed on the brainstem slices from juvenile (6–13 days old) mice expressing EGFP (Slc6a5-EGFP) under the control of the promoter for the neuronal GlyT_2_ (Zeilhofer et al., 2005). Since more than 90% of GlyT_2_ EGFP-positive neurons are immunoreactive for glycine, we consider EGFP-labeled neurons in this study to be putative glycinergic neurons. In order to further dissect the mechanism of muscarinic effects on the preBötC neurons, brainstem slices obtained from M_2_R knockout (M_2_^−/−^) mice (Gomeza et al., 1999), crossed with the Slc6a5-EGFP mice, were used in some experiments. All experiments were conducted in accordance with the Animal Protection Law of Germany and the European Communities Council Directive of November 1986/86/609/EEC and were approved by the District Government.

After anesthesia and decapitation, brains were rapidly removed and immersed in ice-cold high sucrose artificial cerebrospinal fluid (aCSF) containing (in mM): 75 sucrose, 125 NaCl, 3 KCl, 7 MgCl_2_, 1.25 NaH_2_PO_4_, 25 NaHCO_3_, 0.3 CaCl_2_ and 30 D-glucose. Transverse slices (300–350 μm) were cut and transferred into warm aCSF (35°C) for 10 min and kept at room temperature thereafter in normal aCSF for at least 2 h before individual slices were transferred to a recording chamber mounted on the stage of an upright microscope. Brain regions of interest and individual neurons therein were visualized by means of Dodt infrared gradient contrast in conjunction with a contrast-enhanced CCD camera and by fluorescent light. Unless otherwise stated, recordings were performed in normal aCSF containing (in mM): 125 NaCl, 3 KCl, 1.5 CaCl_2_, 1 MgCl_2_, 1.25 NaH_2_PO_4_, 25 NaHCO_3_ and 30 D-glucose at 30°C, using a submerged chamber equipped with a gravity-driven perfusion system. All buffer solutions were constantly bubbled with carbogen (95% O_2_/5% CO_2_) to maintain pH at 7.4. Drugs and chemicals were obtained from Tocris (Cologne, Germany) or Sigma (Deisenhofen, Germany).

### Electrophysiological Recordings and Analysis

**Figure 1:**
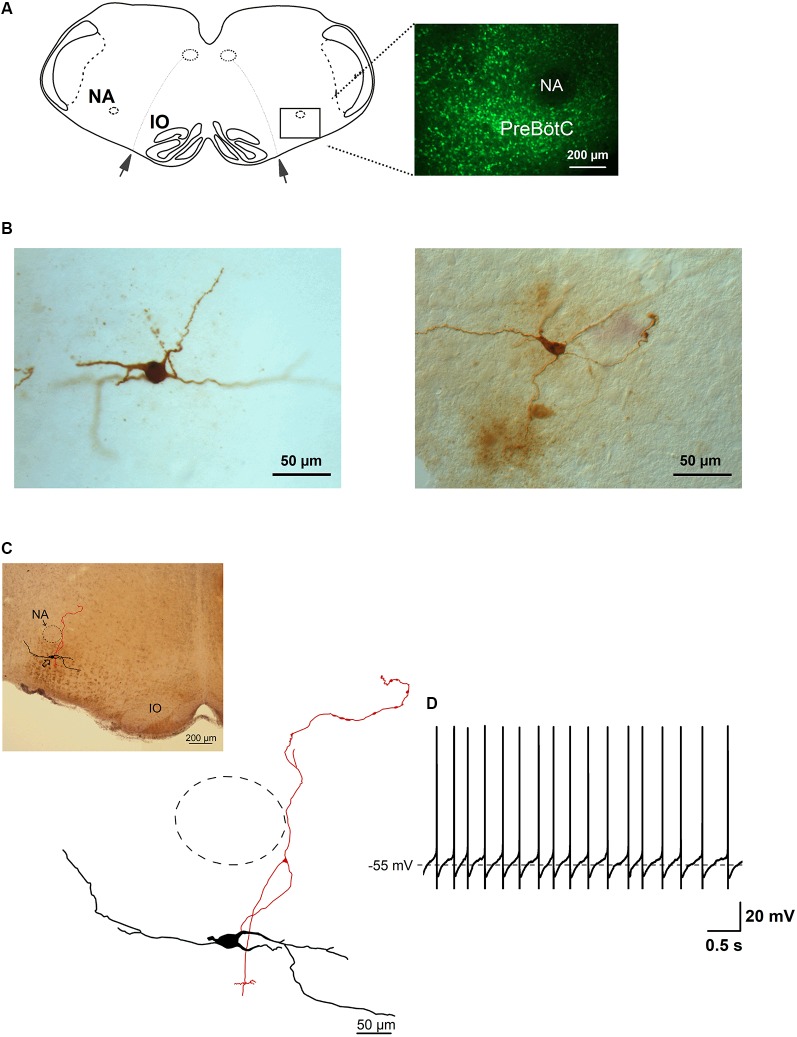
Glycine transporter type 2 (GlyT_2_) enhanced green fluorescent protein (EGFP)-positive neurons in the preBötzinger complex (preBötC). Panel **(A)** is a schematic drawing of a brainstem slice showing the location of the preBötC. Nucleus ambiguus (NA), inferior olive (IO) and the rootlets of n. XII (arrows) are used as landmarks. The fluorescent micrograph (60 μm thick) on the right shows the distribution of EGFP-positive neurons and the location of the preBötC ventral to NA, which itself is free from EGFP label. **(B)** Two examples of light micrographs of biocytin-labeled neurons (60 μm thick sections). Panel **(C)** shows a reconstructed drawing of a biocytin-filled neuron, which is a projection of five consecutive sections (60 μm), re-cut from the slice (350 μm) after electrophysiological recording. The upper-left micrograph illustrates the location of the neuron. The approximate location of NA is indicated by the dashed circle. **(D)** This membrane voltage trace shows the spontaneous discharge pattern of the neuron depicted in **(C)**, recorded in whole-cell current-clamp in physiological external potassium and with GABAergic synaptic inhibition blocked.

The preBötC region was identified based on its location relative to nearby landmarks such as the inferior olive (IO), the hypoglossal nucleus and nerve (XIIn) and nucleus ambiguus (NA), with the help of a mouse brainstem atlas (Ruangkittisakul et al., 2011). The preBötC region corresponds to the area just ventrolateral to the NA (Figure 1A) and neurons located in this area are referred to as “preBötC neurons” in this article. Whole-cell recordings of neuronal firing and excitatory postsynaptic currents (EPSCs) were performed with patch pipettes filled with (in mM): 135 K-gluconate, 5 HEPES, 3 MgCl_2_, 4 NaCl, 5 EGTA, 2 Na_2_ATP and 0.3 Na_3_GTP (pH 7.3). In some experiments, biocytin (1%) was included in the pipette solution for intracellular filling and later morphological analysis. Inhibitory postsynaptic currents (IPSCs) were recorded with pipettes filled with (in mM): 130 CsCl, 3 MgCl_2_, 5 EGTA, 5 HEPES, 2 Na_2_ATP, 0.3 Na_3_GTP and 5 QX-314 (pH 7.3). Resistances of whole-cell patch pipettes in bath ranged from 3 to 5 MΩ. Field potentials were monitored with extracellular recording pipettes filled with modified aCSF, in which bicarbonate was replaced with HEPES to avoid pH changes.

APs were recorded in current-clamp mode in the presence of the GABA_A_ receptor antagonist picrotoxin (100 μM) in the bath solution. Synaptic responses were evoked with constant current pulses (60–200 μA; 0.1 ms in duration) through a bipolar tungsten electrode located in close proximity to the preBötC. Synaptic currents were monitored in voltage-clamp mode with neurons held at −70 mV (after correcting for liquid junction potentials). Series resistance was about 10–25 MΩ and was compensated by 60–80%. EPSCs were pharmacologically isolated with the GABA_A_ receptor antagonist picrotoxin (100 μM) and the glycine receptor antagonist strychnine (10 μM). IPSCs were recorded in the presence of the glutamate receptor antagonist kynurenic acid (KA, 2 mM). For field potential recordings, calcium concentration was raised to 3 mM to enhance evoked synaptic responses, and picrotoxin (100 μM) and strychnine (10 μM) were included in bath media to obtain purely field excitatory postsynaptic potentials (fEPSPs). APs were filtered at 6 kHz and sampled at 20 kHz using an Axoclamp 200 amplifier in conjunction with a Digidata 1200 interface and pClamp9 software (Molecular Devices, Sunnyvale, CA, USA). Synaptic signals were filtered at 2 kHz, sampled at 20 kHz and recorded with a Multiclamp 700B amplifier in conjunction with a Digidata 1440A interface and pClamp 10 software (all from Molecular Devices). Data analysis was performed off-line with Clampfit (Molecular Devices). The negative peaks in the recorded signal were used to determine the amplitudes of synaptic currents/potentials.

### Neuronal Reconstruction and Morphometric Analysis of Biocytin-Filled Neurons

Slices containing biocytin-filled neurons were transferred into 4% paraformaldehyde in 0.1 M phosphate buffer (pH 7.4), fixed overnight, cryo-protected and then re-sectioned at 60 μm thickness on a cryostat. Sections were directly mounted onto poly-L-lysine coated microscope slides, air-dried and stored at −20°C until used for histology. Biocytin-filled neurons were processed for visualization using an avidin-biotin conjugated horseradish peroxidase reaction (ABC standard Elite Kit, PK-4000, Vector Lab). The sections were treated with a mixture of methanol/acetone (7:3) for 10 min, rinsed in PBS and endogenous peroxidase was quenched with 0.5% H_2_O_2_ in PBS for 30 min. After several rinsing steps, the sections were incubated in 0.1% Triton X-100 in PBS for 1 h and then incubated overnight in avidin-biotinylated HRP in PBS and 0.1% Triton X-100. For visualizing the tracer, sections were reacted with diaminobenzidine and hydrogen peroxidase (DAB Kit SK-4100, Vector), followed by a very brief counterstaining with Hematoxylin QS (H-3404, Vector) and directly embedded in Fluoromount. Images were captured, under epifluorescent illumination, using a bandpass filter (450–490 nm) for excitation and a long pass filter (520 nm) for emission. Bright-field images were taken with a digital camera (ColourView II) mounted on a Zeiss Axioscope to locate the biocytin-labeled neurons within the preBötC area.

The biocytin-labeled neurons were reconstructed onto a two-dimensional plane using a camera lucida with the aid of a drawing tube attached to a light microscope (Zeiss). Cell bodies, dendrites, and axons were traced on paper from consecutive 60 μm thick sections using an ×40 objective and the paper drawings were digitized using a PC-based scanner and captured by Neurolucida software (MBF Biosciences, Inc., Williston, VT, USA). Morphological parameters related to the cell body, the dendritic tree, and the axon with its collaterals, were extracted with NeuroExplorer software. Cell body parameters included the perimeter of the cell (length of the contour representing the cell body), area (corresponding to the flat 2D surface occupied by the neuronal soma), compactness and the form factor. Numerical values of somatic compactness ([√(4/π)×area)]/feret max) ranged from 0 to 1. A circle is the most compact shape (compactness = 1), while a square has a compactness of 0.8. The somatic form factor is a measure of circularity and describes how spherical the cell body is (form factor = 4πa/p^2^, where *a* is the soma area and *p* is the perimeter of the soma in the horizontal plane). This value directly reflects the complexity of a somatic perimeter. A higher numerical value of the form factor represents a more complex somatic perimeter. As the contour shape of the cell body approaches that of a perfect circle it tends to a maximum value of 1. In contrast, as the contour shape flattens out, this value approaches a minimum of 0.

**Figure 2:**
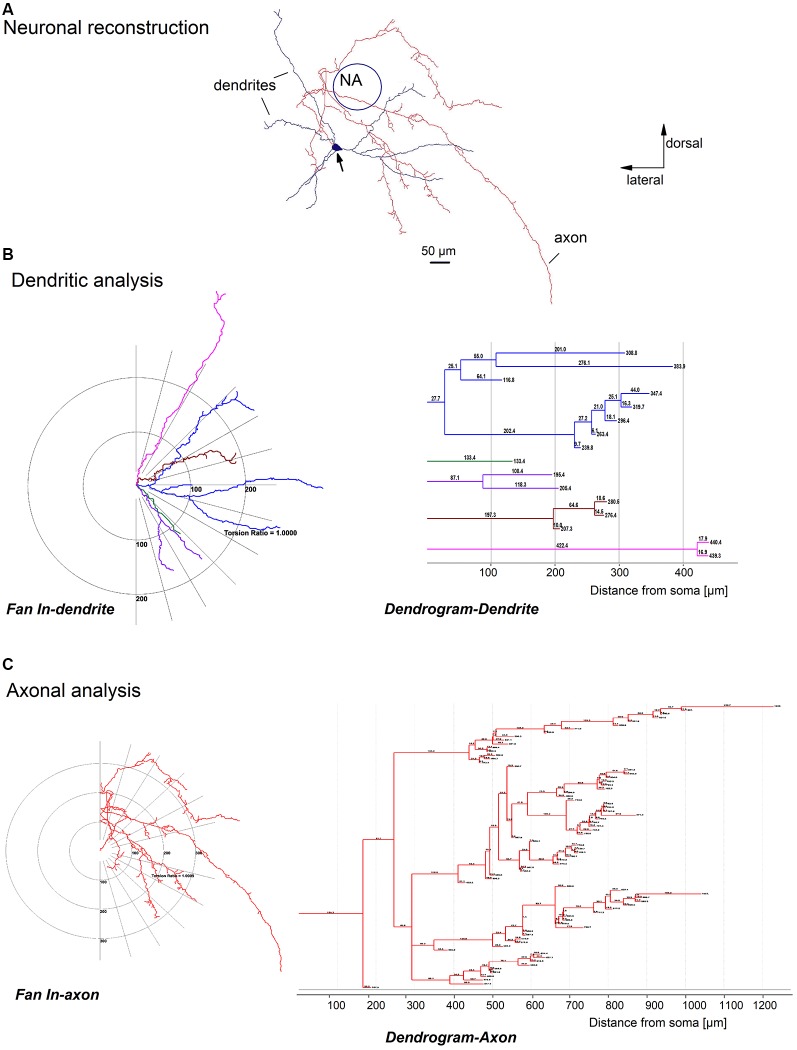
Quantitative analysis of a biocytin-labeled glycinergic preBötC neuron. Panel **(A)** shows an example of a glycinergic preBötC neuron, reconstructed with the help of Neurolucida software, showing the soma (arrow), dendrites (blue) and axons (red). Panels **(B,C)** are schematic projections of the dendrites (*fan in* dendrite) and axons (*fan in* axon) of the neuron illustrated in **(A)**. Note that all dendrites **(B)** and axon collaterals **(C)** on the left diagrams are rotated over to one side and projected on the half-plane. The distances to the soma for each dendrite **(B)** and axonal collateral **(C)** were quantified and plotted as *dendrograms* for the dendrite and axon on the right.

Morphological parameters related to the dendritic tree included: (1) number of first-order dendrites (primary dendrites); (2) dendritic length corresponding to the sum of the length of all segments of the dendritic tree; (3), number of bifurcations reflecting the number of dendritic segments (each bifurcation corresponding to a point of dendritic branching originating two or more dendritic segments); and (4) mean length of the dendrite. Using Branched Structure Analysis (NeuroExplorer) a *dendrogram* was generated to visualize the branching pattern of a dendritic tree of a neuron (Figure 2). We used Sholl analysis to provide further information about the extension of the dendritic tree, such as the “fan in” projection that displays dendritic and axonal processes. The maximal dendritic distance from the soma—derived by Sholl analysis—was the most remote point of the dendritic tree of the cell. This type of analysis of the dendritic tree was also applied to obtain the axon length and the axonal arborization pattern. To visualize reconstructed biocytin-filled GlyT_2_-EGFP labeled neurons, the images were transferred into Adobe Photoshop CS3, extracted, and digitally moved into the micrograph of the neighboring histological slice after proper scaling as demonstrated in Figure 1C.

### Statistical Analysis

Data are expressed as mean ± SEM. Statistical comparisons of data were performed using Student’s *t*-test or analysis of variance (ANOVA), as appropriate. Significance was assumed for *p* < 0.05.

## Results

### Location and Morphology of Glycinergic Neurons in the PreBötC

We recorded the discharge patterns of a total of 85 green GlyT_2_-EGFP-labeled putative glycinergic neurons in brainstem slices from P6 to 13 mice. The labeled neurons were distributed evenly within the preBötC region (Figure 1A). Figure 1B illustrates two examples of biocytin-filled glycinergic neurons, re-sectioned to 60 μm from the original slice (350 μm) and visualized with the ABC procedure (see “Materials and Methods” section). Soma shape and size, as well as neurites, could be clearly discerned in these sections. An example of a serially reconstructed biocytin-labeled neuron is shown in Figure 1C. Its location within the preBötC region is shown in the corresponding bright-field micrograph. The trace in Figure 1D depicts a whole-cell recording of the spontaneous regular firing pattern of this neuron.

**Figure 3:**
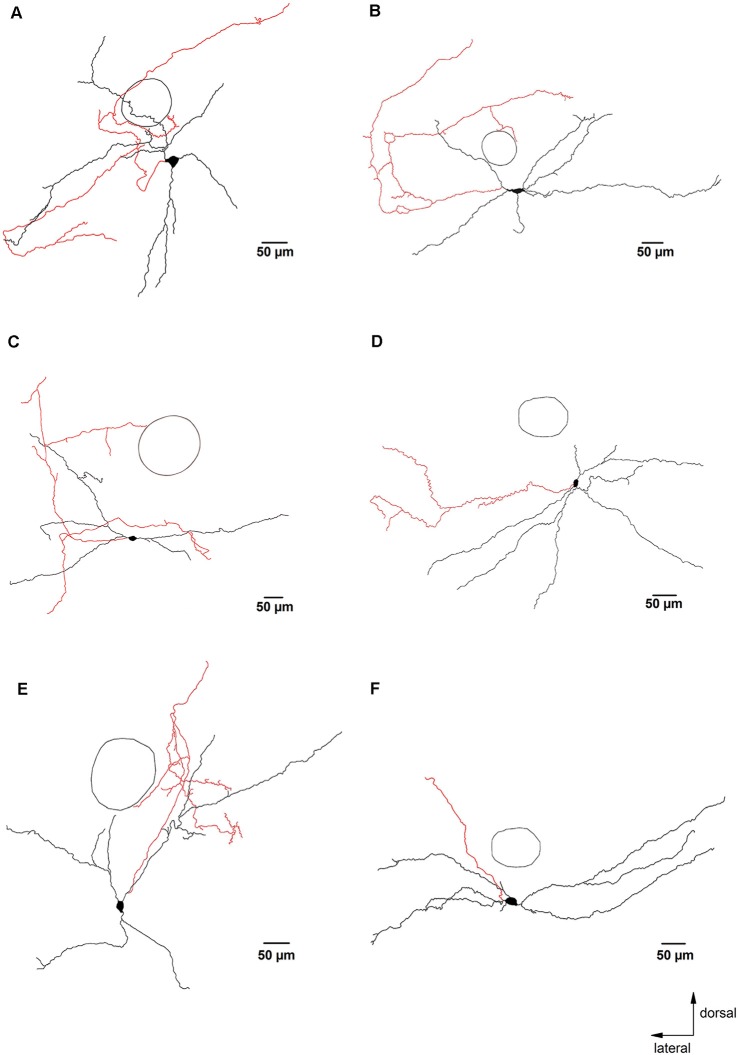
Glycinergic neurons with axons that remained close to the preBötC. Panels **(A–F)** are examples of camera lucida drawings of biocytin-labeled glycinergic neurons in the preBötC. These neurons had axons and axon collaterals that remained mostly within 500 μm from the soma. The circle indicates the location of NA as an orientation reference. The axons are depicted in red.

**Figure 4:**
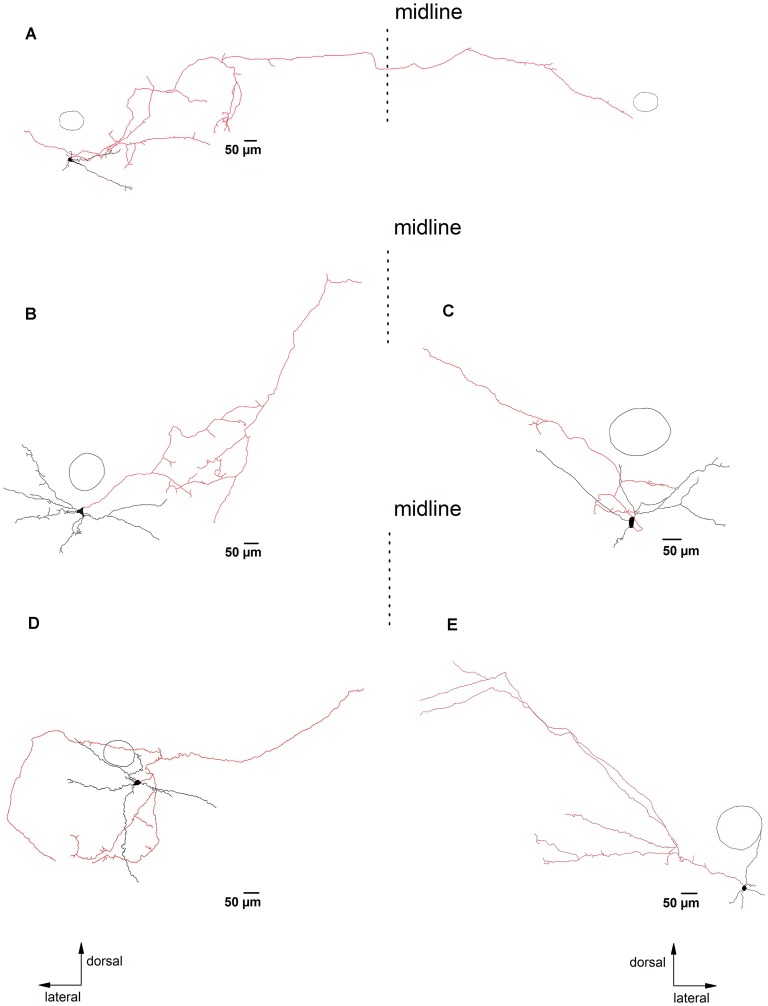
PreBötC glycinergic neurons with long axonal projections. Panels **(A–E)** illustrate biocytin-filled putative glycinergic neurons with axons that project more than 500 μm away from the soma. The dashed lines indicate the midline of the slice. Neurons in **(A,B,D)** were located on the left side, while neurons in** (C,D)** were from the right side of the slice. The reconstructed axons are depicted in red. The circle indicates the location of NA as an orientation reference. Note that the neuron in **(A)** projects to the contralateral preBötC. The other four neurons project in the direction of the hypoglossal nucleus (n. XII).

Since a comprehensive morphometric atlas of glycinergic neurons in the preBötC is still lacking, we performed a detailed morphological analysis of 19 serially-reconstructed, biocytin-filled neurons (Figure 2). Cell bodies were round, triangular or ellipsoidal in shape, and at least three dendrites emerged from the cell body (Figures 1–4). The average soma size was 280 ± 20 μm^2^ (ranging from 120 to 610 μm^2^) and the perimeter was 71 ± 3 μm (from 44 to 117 μm; *n* = 19). The values for largest diameter of neuronal soma were 27 ± 1 μm (from 17 to 44 μm) and 16 ± 1 μm (from 10 to 27 μm) for the shortest diameter. The heterogeneity of shapes and forms was further reflected in a compactness factor which ranged from 0.40 to 0.87 (mean = 0.71 ± 0.02) and roundness which varied from 0.17 to 0.75 (0.52 ± 0.03). The complexity of the somatic perimeter was also considerable, with a form factor between 0.32 and 0.93 (0.73 ± 0.04).

The reconstructed glycinergic neurons had 3–8 primary dendrites (4.2 ± 0.3), and the dendrites bifurcated at various distances from the soma, with 2–22 bifurcating nodes (mean = 7.8 ± 1.0; Figures 2–4). A typical analysis for the dendritic tree is illustrated in Figure 2B, showing a schematic representation of the dendrites with a *fan-in* projection on the left (projected onto the half-plane) and the extracted *dendrogram* on the right to visualize the branching pattern; the latter was used for further quantification. The majority of the neurons (15 out of 19) had dendrites remaining within a distance of 500 μm from the soma. The total dendritic length was 1,850 ± 100 μm (from 1,100 to 2,500 μm; *n* = 19). The mean dendritic segment length was 480 ± 30 μm (from 260 to 700 μm; cut-off dendrites were excluded from the analysis). In general, the dendrites were smooth, but occasionally we observed short string-like appendages resembling stubby or thin spine-like structures on individual dendrites (*n* = 4).

Axons were identified as smaller diameter processes that typically (13 out of 19 neurons) arose directly from the soma. In some cases, the primary axon originated from one of the primary dendrites, at a short distance from the soma (13 ± 3 μm, *n* = 6). Examples of such dendritic-origin axons are depicted in Figures 1C, 3B,E, with axons that originated at 6 μm, 27 μm and 17 μm from the soma, respectively. Axons were mostly smooth, with axonal swellings seen infrequently at their distal ends (Figure 1C, camera lucida drawing). We applied Sholl analysis to the axonal arborization patterns with *fan-in* projections and a dendrogram (Figure 2C). Except for one case shown in Figure 3F, the axons bifurcated at various distances from the soma. The axons gave off 2–73 collaterals (14 ± 4) ending at a maximal distance from the soma of 200–3,000 μm (940 ± 160 μm). The axons had on average a total length of 2,100 ± 400 μm (from 250 to 6,200 μm) with a mean segmental length of 700 ± 70 μm (two cut-off axons were excluded from the analysis of axon length).

Most reconstructed glycinergic neurons (13 out of 19) had axons which remained within their own dendritic field. The remaining 6 neurons had axons that branched out beyond their dendritic area. One neuron had an axon that ran ventromedially (Figure 2), another neuron had an axon that crossed the midline and ran towards the contralateral preBötC (Figure 4A), while the other four neurons projected towards the XIIn (Figures 4B–E). Clear morphological differences appeared when we sub-grouped the neurons according to the length of the axons from the soma (see below). For grouping purposes, we named neurons with axons that stayed close to the preBötC (less than 500 μm; Figure 3) “local” neurons, while neurons with axons projecting more than 500 μm away from the preBötC (Figure 4) were named “projecting” neurons. Using this classification scheme to divide the neurons into two groups, we found that several of the parameters for axon and dendritic branching differed significantly between the two groups, despite similar somatic parameters (Table 1). The maximal axonal distance from the soma in projecting neurons was much longer, they had significantly more bifurcations, longer mean length of axons, and greater total axon length than local neurons. The projecting neurons had a more compact dendritic tree as evidenced by a shorter maximal dendritic distance and a smaller mean dendritic length (Table 1). Although the number of dendritic bifurcations was similar in local and projecting neurons, the latter had 30% more primary dendrites. Occasionally (two local and two projection neurons), stubby/thin spine-like appendages were observed on individual dendrites.

**Table 1:** Quantification of the morphology of glycinergic neurons in the preBötC.

Parameters	Local neurons	Projecting neurons
	(*n* = 13)	(*n* = 6)
**Somatic**		
Perimeter (μm)	69 ± 5	77 ± 8
Soma area (μm^2^)	280 ± 33	297 ± 26
Form factor	0.74 ± 0.04	0.68 ± 0.09
Compactness	0.72 ± 0.03	0.73 ± 0.03
**Dendritic**		
Number of primary dendrites	3.7 ± 0.4	5.2 ± 0.4*
Number of bifurcations	7.0 ± 0.7	9.5 ± 2.8
Total dendritic length (μm)	1,800 ± 130	1,900 ± 200
Mean length of dendrites (μm)	530 ± 38	370 ± 23*
Maximal dendritic distance from	396 ± 29	292 ± 24*
soma (μm)
**Axonal**		
Number of bifurcations	5.7 ± 1.9	33 ± 9*
Total axonal length (μm)	1,100 ± 230	4,000 ± 600*
Mean length of axons (μm)	570 ± 72	960 ± 110*
Maximal axonal distance from	355 ± 31	1,200 ± 260*
soma (μm)

Biocytin-filled EGFP-positive neurons were divided into two groups, according to whether their axons stayed close (within 500 μm) to the soma (i.e., “local” neurons) or projected more than 500 μm away from the soma (“projecting” neurons). Values are given as mean ± SEM. *p < 0.05, a significant difference between the two groups.

### Electrophysiological Profiles of PreBötC Glycinergic Neurons

**Figure 5:**
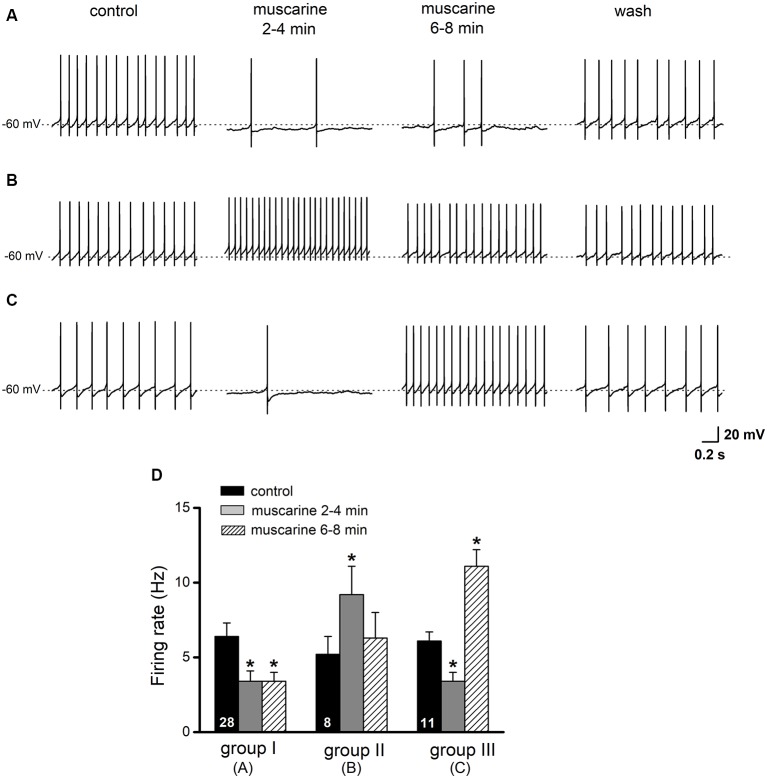
Effects of muscarine on regular spiking glycinergic neurons in the preBötC. Current-clamp recordings of spontaneously active neurons were made in the presence of picrotoxin (100 μM). Original traces in **(A–C)** illustrate examples of the three different response patterns of preBötC glycinergic neurons to muscarine application (10 μM). Scale bar in **(C)** also applies for traces in **(A,B)**. Histograms in **(D)** summarize the early (2–4 min) and late (6–8 min) effects of muscarine on firing rate. Groups are based on the responses shown in **(A–C)** and are further described in the “Results” section. The numbers inside the columns indicate the number of neurons of the respective groups. *p < 0.05, compared to respective control.

**Figure 6:**
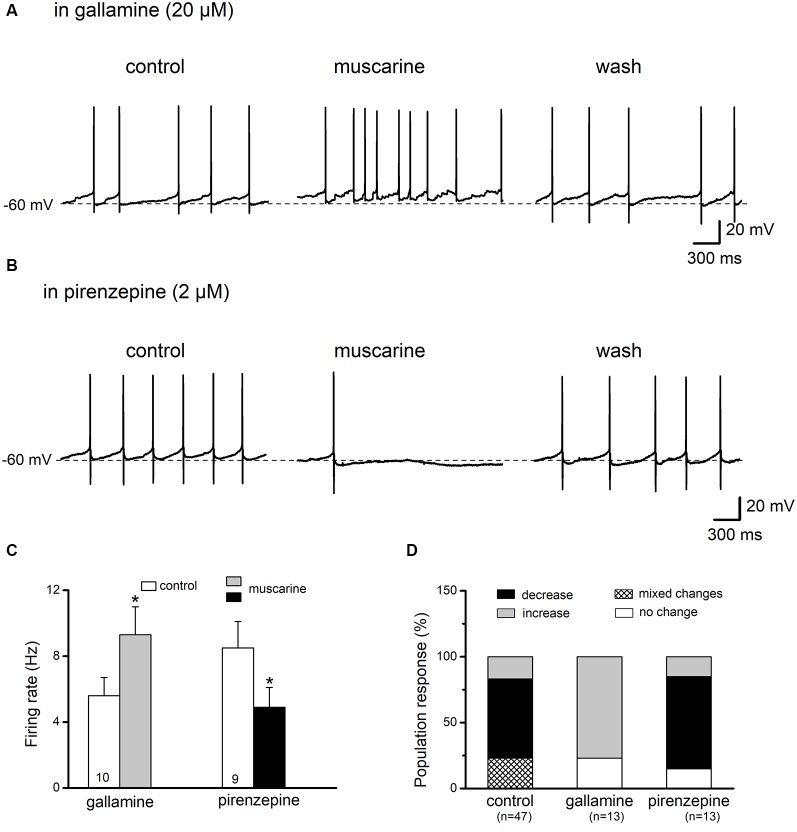
Pharmacological profiles of muscarinic effects on preBötC glycinergic neurons. Panels **(A,B)** are illustrations of the effects of muscarine (10 μM for 6–8 min) in the presence of the M_2_R antagonist gallamine (20 μM) and of the M_1_R antagonist pirenzepine (2 μM), respectively. Muscarine effects on firing were partially reversible (wash). The histogram in **(C)** summarizes responses to muscarine in control and in the presence of muscarinic receptor blockers. The histogram in **(D)** summarizes responses to muscarine. Mixed response patterns to muscarine were observed in the control group (with muscarine alone), with most neurons (28 out of 47 neurons or 60%) showing a reduction in firing rate. The majority of responses were shifted to an increase in firing rate in the presence of gallamine (10 out 13, or 77%) or a decrease in the presence of pirenzepine (9 out 13, or 70%). *p < 0.05, compared to respective control.

**Figure 7:**
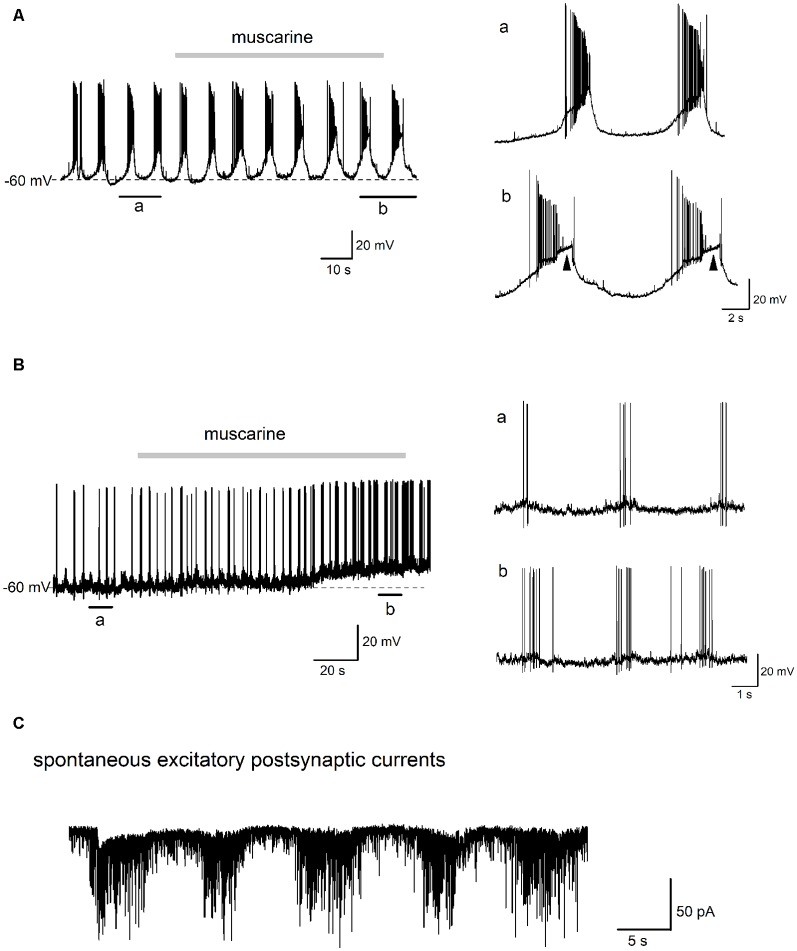
Effect of muscarine on the “burst-like” firing glycinergic neurons in the preBötC. Traces in **(A)** show the effects of muscarine (10 μM) recorded in a “burst-like” glycinergic neuron. This cell discharged in a rhythmic “burst-like” pattern consisting of alternating bursts of action potentials (APs) interspersed with strong membrane hyperpolarization and cessation of firing. Two “bursts” of rhythmic discharges before and during muscarine (indicated by black bars underneath the trace) are enlarged for closer inspection on the right. Note that muscarine had such a strong excitatory effect on this neuron that it induced a depolarization block during the burst (arrowheads). Traces in **(B)** are from a different rhythmically firing “burst-like” neuron****, whose morphology is shown in Figure 1B (right). This neuron fired brief, rhythmic “bursts” of 3–5 APs under control conditions as illustrated on a larger time scale on the right. Note the lack of a large depolarizing envelope as in panel **(A)**. Muscarine application depolarized this neuron and increased the burst frequency and number of spikes per burst. Trace in **(C)** is from an EGFP-positive glycinergic neuron recorded in voltage-clamp mode at −70 mV. Note the rhythmic bursts of spontaneous excitatory postsynaptic currents (EPSCs), which were recorded in the presence of picrotoxin (100 μM) and strychnine (10 μM) to block fast GABA- and glycinergic inputs, respectively.

Input resistance and membrane capacitance of preBötC glycinergic neurons varied considerably from 134 MΩ to 1,200 MΩ (410 ± 21 MΩ) and from 19 pF to 97 pF (51 ± 2 pF), respectively (*n* = 85). Under current-clamp mode, these neurons were spontaneously active under our recording conditions with a GABA_A_ receptor antagonist in the bath. Most of them (*n* = 80) fired regular repetitive APs at a frequency of 14.6 ± 0.7 Hz (from 1.3 Hz to 28.4 Hz; Figures 1D, 5, 6). Five neurons fired in a “burst-like” pattern, with clusters of APs riding on top of a prolonged membrane depolarization followed by an inter-burst hyperpolarization (Figures 7A,B). The “burst-like” neurons had 12 ± 4 bursts per minute on average, with a mean inter-burst interval of 7 ± 4 s (*n* = 5). Each burst consisted of 3–20 APs (10 ± 3), and the bursts duration was 1.2 ± 0.8 s. When voltage-clamped at a holding potential of −70 mV, a similar percentage of preBötC glycinergic neurons (2 out of 25) displayed rhythmic bursts, consisting of a barrage of EPSCs (Figure 7C). This finding demonstrates that the bursting activity in these neurons was network-driven.

No correlation was found between the mode of firing and axonal projection patterns. Among the above-mentioned 19 reconstructed EGFP-positive neurons, 18 neurons were regular-spiking and one neuron discharged in rhythmic “bursts.” The input resistance (446 ± 66 MΩ) and the membrane capacitance (54 ± 4 pF) of the reconstructed neurons were within the range of values derived for the cell population as a whole. Most regular-spiking neurons (*n* = 12) had axons that remained close to preBötC (i.e., they were “local” neurons), while the others (*n* = 6) had axons projecting more than 500 μm away from preBötC (classified as “projecting” neurons), suggesting that there exists no correlation between the mode of firing in the slice and the axonal projecting pattern.

### Responses of PreBötC Glycinergic Neurons to Muscarine

The presence of cholinergic muscarinic receptors in the preBötC as well as the local modulation of respiratory function by muscarine has been well established (Shao and Feldman, 2000, 2005; Lai et al., 2001; Zanella et al., 2007; Koch et al., 2013). We investigated the actions of bath-applied muscarine (10 μM) on AP firing of glycinergic neurons, held around −60 mV (as read from the average inter-spike membrane potential) by injection of steady hyperpolarizing DC current (from −10 to −50 pA). As illustrated in Figures 5A–C, we observed three different responses in a total of 47 neurons tested with muscarine: a decrease in firing rate (*n* = 28, 59.6%, group A), an increase (*n* = 8, 17.0%, group B), or a decrease followed by an increase (*n* = 11, 23.4%, group C). In group A neurons, muscarine decreased firing rate from 6.4 ± 0.9 Hz to 3.4 ± 0.6 Hz (*p* = 0.001, paired *t*-test), while in group B neurons, muscarine increased the firing rate from 5.2 ± 1.3 Hz to 9.2 ± 1.9 Hz (*p* = 0.013, paired *t*-test). In the 11 neurons with mixed muscarinic responses (group C), an initial slowing of the firing rate from 6.1 ± 0.6 Hz to 3.4 ± 0.6 Hz (*p* = 0.001) was followed by a large increase to 11.1 ± 1.2 Hz (*p* = 0.001) before recovering to control level upon washout (5.3 ± 0.6 Hz; Figure 5D). The muscarine-induced changes in spike firing were accompanied by corresponding shifts in membrane potentials, i.e., hyperpolarization with reduced firing and depolarization with increased firing (Figures 5A–C).

Again, there was no correlation between the effects of muscarine on neuronal firing and the axonal projection patterns. Thirteen of these 47 regular-spiking neurons tested with muscarine were fully reconstructed. Consistent with the prevailing suppressive effect of muscarine, most reconstructed neurons (*n* = 10) displayed reduced firing in response to muscarine, one neuron increased its firing rate and two other neurons showed mixed responses. The reconstructed neurons in Figures 1C, 2, 3A,B,E,F, 4A were among cells that showed a slowing of firing rate in response to muscarine, illustrating the heterogeneous morphological appearance of these reduced-firing neurons. They were characterized by either small or elaborated dendritic trees (Figures 1C, 3E, respectively), simple axons (Figure 3F), or complicated axons with many collaterals forming a complex axonal network remaining in close vicinity of the soma (Figure 3A) or projecting away from the cell (Figure 4A). Using our selection criteria, 7 out of 10 neurons were “local” neurons, while the remaining three neurons sent their axons to the contralateral preBötC (Figure 4A) or ventromedial/dorsomedial regions and were therefore classified as “projecting” neurons. The interesting spatial pattern of axon collaterals in Figure 4D was from the neuron with muscarine-induced excitation. This neuron had major axon collateral that extended dorsomedially away from the preBötC, but it also collateralized extensively within the dendritic field in the preBötC, and thus might be a mixed “local/projection” neuron.

### Pharmacological Profiles of Muscarinic Responses in PreBötC Glycinergic Neurons

Members from both the M_1_ type (M_1_/M_3_/M_5_) and M_2_ type (M_2_/M_4_) receptor families, classified according to their coupling with G_q/11_ and G_i/o_, respectively (Wess, 1996; Caulfield and Birdsall, 1998), are present in the preBötC (Lai et al., 2001). Therefore, we employed the respective antagonists to determine the mechanisms behind the observed muscarinic modulation of glycinergic neuron firing in this area of the brainstem. In the presence of the M_2_R-preferring antagonist gallamine (20 μM), preBötC glycinergic neurons responded to muscarine (10 μM) with either an increase in firing rate (from 5.6 ± 1.1 Hz to 9.3 ± 1.7 Hz, *n* = 10 out of 13 or 76.9%; *p* = 0.001) or no change (*n* = 3; Figures 6A,C,D). In contrast, most neurons tested in the presence of the M_1_R-preferring antagonist pirenzepine (2 μM) showed a decrease in firing rate in response to muscarine application (from 8.5 ± 1.6 Hz to 4.9 ± 1.2 Hz, *n* = 9 out of 13 or 69.2%; *p* = 0.001), while a minority displayed either no change (*n* = 2) or an increase in firing (*n* = 2; Figures 6B–D). These results indicate that the muscarinic inhibitory and excitatory effects on the firing of preBötC glycinergic neurons are mediated by M_2_Rs and M_1_Rs, respectively.

We next tested the effect of muscarine on “burst-like” glycinergic neurons in the preBötC. As shown in the example in Figure 7A, this “burst-like” neuron fired 6–8 bursts per min (7.0 ± 0.2, measured over 3 min before muscarine application) and each burst consisted of a series of APs (20.0 ± 3.0 per burst), followed by robust inter-burst membrane hyperpolarization. Muscarine (10 μM) prolonged the average duration of the burst (from 2.3 ± 0.1 s to 3.3 ± 0.2 s) and lengthened the inter-burst interval (from 6.6 ± 0.3 s to 7.7 ± 0.1 s). The magnitude of the depolarizing envelope underlying the burst was greatly increased, and so much so, that a depolarization block was observed in each burst from the second minute onwards after the start of the bath application of muscarine (arrowheads on the right). Another “burst-like” neuron showed a different rhythmic firing pattern (Figure 7B). This pattern consisted of the spontaneous firing of short, but frequent bursts (16 bursts per minute, 3.6 ± 0.4 APs per burst, burst duration 0.4 ± 0.1 s, averaged from consecutive bursts in a 2 min control period before muscarine). In contrast to the neuron in Figure 7A, this neuron had a smaller depolarizing envelope and a relative small inter-burst hyperpolarization. Muscarine (10 μM) had a clear excitatory effect on this neuron, manifested by a prolongation of burst duration (from 0.4 ± 0.1 s to 1.4 ± 0.2 s) and an increase in the number of APs per burst (from 3.2 ± 0.4 to 7.2 ± 0.4). The number of bursts per min was slightly increased (from 16 to 20) and the inter-burst interval became shorter (from 3.1 ± 0.3 s to 2.5 ± 0.3 s). These changes in the bursting pattern were accompanied by an overall membrane depolarization after muscarine (Figure 7B).

### Muscarine Suppresses Synaptic Inputs to PreBötC Neurons

**Figure 8:**
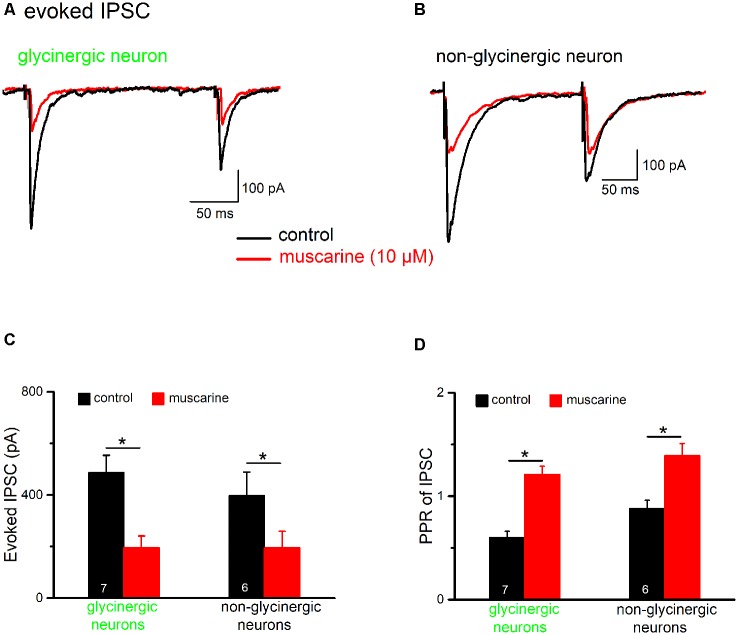
Muscarine reduces inhibitory postsynaptic currents (IPSCs) in preBötC neurons. Voltage-clamp recordings of evoked IPSCs were made in the presence of kynurenic acid (2 mM). Cells were held at −70 mV and IPSCs are shown as downward deflections due to the high chloride-containing internal solution. Traces in **(A,B)** are example traces from an EGFP-positive (“glycinergic”) and an EGFP-negative (“non-glycinergic”) neuron in the preBötC, respectively. Pairs of IPSCs were evoked by delivering two identical, electrical stimuli at half-maximal amplitude to the slice, 200 ms apart, using a nearby placed stimulating electrode. Note the decrease in peak IPSC amplitude in response to the 2nd stimulus compared to the 1st in control perfusate. Traces recorded before and after bath application of muscarine (10 μM) are superimposed. The paired-pulse ratio (PPR) was determined by dividing the peak amplitude of 2nd IPSC by the peak amplitude of the 1st IPSC. A PPR < 1 indicates paired-pulse depression (PPD), while a PPR > 1 signifies paired-pulse facilitation (PPF). The muscarine-induced inhibition of 1st IPSC and the accompanying change in PPR were quantified, and group means values and SEM measured for these parameters in glycinergic and non-glycinergic neurons are shown in the histograms in **(C,D)**, respectively (*p < 0.05).

Since preBötC glycinergic neurons themselves are inhibited by other glycinergic neurons during inspiration (Winter et al., 2009) and could thereby dynamically tune the preBötC network (Sherman et al., 2015; Fortuna et al., 2019), we next examined the impact of muscarinic modulation on GABAergic/glycinergic synaptic inhibition in preBötC neurons. IPSCs were evoked by electrical pulses delivered using a bipolar stimulating electrode placed in the slice near the recorded cell and in the presence of the ionotropic glutamate receptor antagonist kynurenic acid in the bath (2 mM). In order to identify the potential site of drug action (pre- vs. post-synaptic), we used a paired-pulse protocol in which two identical stimuli were given 200 ms apart (Figures 8A,B). The paired-pulse ratio (PPR), obtained by dividing the amplitude of 2nd IPSC by that of 1st IPSC, was 0.60 ± 0.06 (*n* = 7), indicating that these synapses display a marked paired-pulse depression (PPD) under control conditions. Muscarine (10 μM) greatly reduced the amplitude of the 1st IPSC (from 484.7 ± 68.2 pA to 195.7 ± 45.7 pA, paired *t*-test, *p* = 0.0002; i.e., a reduction of 61.3 ± 3.7%), and increased the PPR from 0.60 ± 0.06 to 1.21 ± 0.08 (paired *t*-test, *p* = 0.0007, *n* = 7; Figures 8C,D, left columns). These changes in the PPR suggest a presynaptic site of action of muscarine. However, in contrast to the well-established role of muscarinic M_2_Rs in presynaptic inhibition at various central synapses including those of hypoglossal motoneurons (Bellingham and Berger, 1996; Pagnotta et al., 2005), a virtually identical suppression of evoked IPSCs was observed in preBötC glycinergic neurons when muscarine was applied in the presence of M_2_R-preferring antagonist gallamine (20 μM, *n* = 3, reduction of 62.7 ± 5.6%). To determine whether muscarine-induced suppression of IPSCs is a generalized phenomenon in the preBötC, we applied the drug also to a group of un-labeled (presumably non-glycinergic) neurons (Figure 8B). Under control conditions, these cells displayed less PPD than the glycinergic ones (PPR 0.91 ± 0.09, *n* = 6; *p* = 0.016). Muscarine reduced the first IPSC from 397.7 ± 91.0 pA to 195.3 ± 64.5 pA (*n* = 6, paired *t*-test, *p* = 0.003; i.e., a decrease of 56.2 ± 7.6%) and increased the PPR from 0.91 ± 0.09 to 1.51 ± 0.16 (paired *t*-test, *p* = 0.004; Figures 8C,D), similar to what we found in glycinergic neurons.

**Figure 9:**
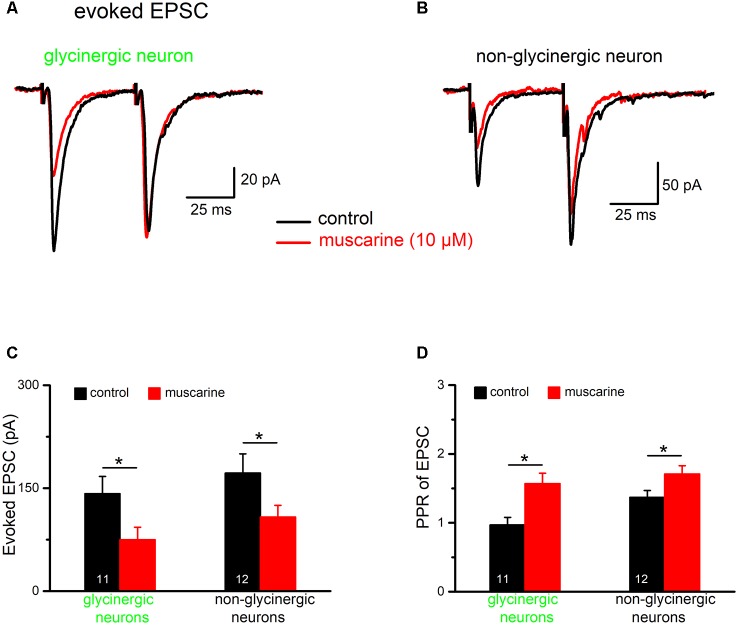
Muscarine suppresses EPSCs in preBötC neurons. Representative voltage-clamp recordings of pairs of evoked EPSCs (stimulus interval was 50 ms) made in the presence of picrotoxin (100 μM) and strychnine (10 μM) in the bath. **(A,B)** Superimposed traces recorded before and during bath application of muscarine (10 μM) in two types of preBötC neurons. Note that in control perfusate, the glycinergic neuron showed PPD while the non-glycinergic neuron showed PPF. Muscarine reduced the amplitude of the 1st EPSC in both groups of neurons as summarized in the histograms shown in **(C)**. The graph in **(D)** summarizes muscarinic effects on PPR of EPSC in the two groups of neurons. This figure shows that muscarine turned the prevailing PPD (PPR < 1) in glycinergic neurons into PPF (PPR > 1; *p < 0.05).

Next, we examined the effect of muscarine on excitatory synaptic transmission onto both glycinergic and non-glycinergic neurons, in the presence of the GABA_A_ receptor antagonist picrotoxin (100 μM) and the glycine receptor antagonist strychnine (10 μM). It is interesting to note that, in control conditions, most non-glycinergic preBötC neurons exhibited paired-pulse facilitation (PPF) of evoked EPSCs (10 out of 12 neurons, see example in Figure 9B; stimulus interval = 50 ms), while only a minority of glycinergic neurons (2 out of 11 neurons) displayed PPF. The remaining glycinergic neurons exhibited either PPD (*n* = 5; Figure 9A) or no change (*n* = 4). As shown in Figure 9C, the amplitude of the 1st EPSC was uniformly suppressed by muscarine (10 μM) in both types of neurons. The reduction in 1st EPSC amplitude was 47.8 ± 5.2% in glycinergic neurons (*n* = 11 from control 141.5 ± 24.7 pA to 75.3 ± 18.2 pA; paired *t*-test, *p* = 0.0002) and 38.1 ± 3.4% in non-glycinergic neurons (*n* = 12 from control 172.9 ± 28.9 pA to 107.8 ± 17.5 pA; paired *t*-test, *p* = 0.0007). Compared to the muscarinic inhibition of IPSCs illustrated in Figure 8C, the muscarinic effect on EPSCs was significantly smaller in both types of neurons (*p* = 0.045 for glycinergic neurons; *p* = 0.020 for non-glycinergic neurons). The muscarine-induced decrease in 1st EPSC amplitude was more pronounced than that of the 2nd EPSC in the paired-pulse protocol resulting in an increase in PPR in both types of neurons (*n* = 11 glycinergic neurons, from a control value of 0.97 ± 0.11 to 1.57 ± 0.15, *p* = 0.023, paired *t*-test; *n* = 12 non-glycinergic neurons, from control 1.37 ± 0.10 to 1.71 ± 0.12, *p* = 0.016, paired *t*-test; Figure 9D), suggesting again a presynaptic site of action for mAChR modulation of excitatory synaptic transmission, although we cannot exclude the contribution of a postsynaptic component (Seeger and Alzheimer, 2001).

### Muscarinic Receptor Subtype-Specific Control of Excitatory Synaptic Drive onto Different Types of PreBötC Neurons

**Figure 10:**
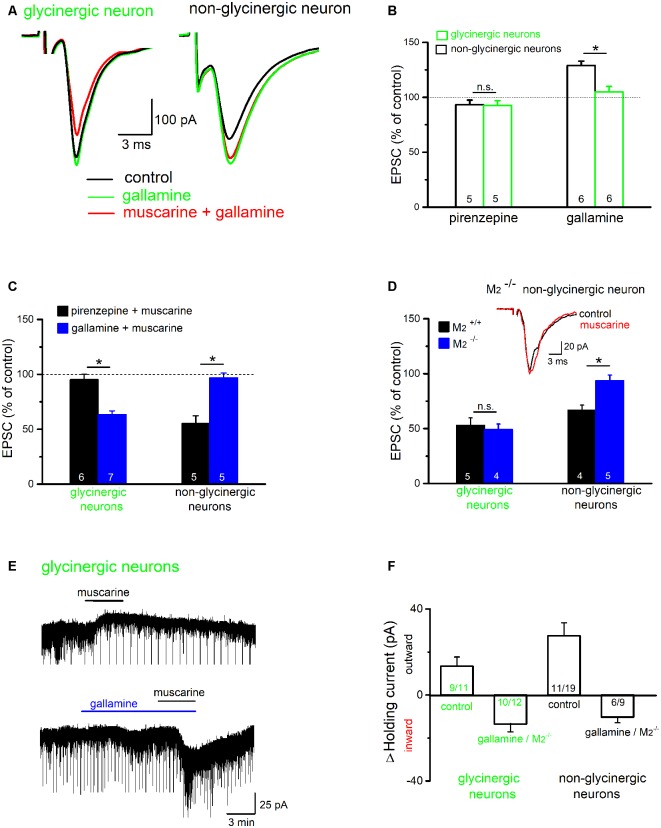
Muscarine exerts a receptor subtype-specific control over excitatory synaptic input onto preBötC neurons. **(A)** Superimposed traces show the effects of the M_2_R blocker gallamine (20 μM) on evoked EPSCs in representative traces from both groups of neurons. Gallamine increased EPSC amplitude and blocked the effect of muscarine (10 μM) in the non-glycinergic neuron (right), but not in the glycinergic neuron (left). **(B)** Histogram summarizes the relative changes in EPSC amplitudes compared to control (amplitude of control EPSC was set to 100%) in response to the application of the M_1_R antagonist pirenzepine (1 μM) or the M_2_R antagonist gallamine. Note that an endogenous M_2_R-mediated tonic inhibition was only observed for EPSCs recorded in non-glycinergic neurons. **(C,D)** Histograms summarize the receptor subtype selectivity of muscarinic modulation of EPSCs in preBötC neurons. Muscarine was applied in the presence of muscarinic acetylcholine receptor (mAChR) antagonists **(C)** or to slices from M_2_ knockout mice (M_2_^−/−^) and their wild-type littermates (M_2_^+/+^; **D**). EPSC amplitude before muscarine application served as control and was set to 100%. Superimposed traces (inset in **D**) from an M_2_^−/−^ slice shows that muscarinic inhibition of EPSC amplitude in non-glycinergic neurons is M_2_R-mediated. **(E)** Muscarinic effects on holding current in glycinergic neurons voltage-clamped at −70 mV. The application of muscarine produced an outward current (upper trace). In the presence of gallamine, we observed an inward current response to muscarine (lower trace). The downward events along the traces are the simultaneously recorded evoked/spontaneous EPSCs. The histogram in **(F)** summarizes shifts in holding current for glycinergic and non-glycinergic neurons under control conditions and with M_2_Rs pharmacologically blocked (gallamine) or genetically disrupted (M_2_^−/−^). The numbers in columns indicate sample number in each group (*p < 0.05, n.s. not significant).

To further identify the receptor subtype involved in muscarinic suppression of excitatory synaptic transmission in the preBötC, we used the M_2_R-preferring antagonist gallamine and the M_1_R-preferring antagonist pirenzepine. A noteworthy finding is that gallamine unmasked an apparent endogenously active, M_2_R-mediated inhibitory muscarinic tone in the brainstem slice, as previously observed in the hippocampus (Seeger et al., 2004; Zheng et al., 2012). In six non-glycinergic preBötC neurons tested, bath application of gallamine alone (20 μM) increased the amplitude of evoked EPSCs from 191.1 ± 52.6 pA to 242.8 ± 63.6 pA (*p* = 0.007, paired *t*-test; 129.3 ± 3.5% of control; Figures 10A,B), and caused a notable inward shift in the holding current of 20.0 ± 4.4 pA (*n* = 6). By contrast, in 6 glycinergic neurons tested, gallamine failed to increase EPSCs in five of the six neurons (105.4 ± 5.1% of control, *n* = 6; Figures 10A,B), despite an appreciable inward shift in holding current (8.8 ± 3.2 pA, *n* = 6). The M_1_R antagonist pirenzepine (1 μM) alone had no appreciable effect on either holding current or the amplitude of EPSCs in both types of neurons (*n* = 5 non-glycinergic neurons, 93.2 ± 4.3% of control; *n* = 5 glycinergic neurons, 92.7 ± 4.4% of control; Figure 10B).

Gallamine abolished the muscarine-induced suppression of EPSCs in non-glycinergic neurons (96.8 ± 4.4% of control, *n* = 5), but not that in non-glycinergic neurons (63.4 ± 3.4% of control, *n* = 7; Figure 10C). Vice versa, pirenzepine (1 μM) almost completely blocked muscarinic suppression of EPSCs in glycinergic neurons (95.2 ± 4.8% of control, *n* = 6), but failed to do so in non-glycinergic neurons (55.2 ± 7.1% of control, *n* = 5; Figure 10C). These observations strongly suggest that muscarine exerts a receptor subtype-specific control of excitatory synaptic drive onto different types of preBötC neurons. To further substantiate this unexpected finding, we used M_2_R knockout mice. Consistent with the pharmacological evidence, muscarine (10 μM) inhibited EPSCs in non-glycinergic neurons from M_2_^+/+^ mice (*n* = 4 from four mice, 66.9 ± 4.5% of control), but failed to do so in M_2_^−/−^ mice (*n* = 5 from four mice, 93.8 ± 5.0% of control; *p* = 0.005; Figure 10D). By contrast, muscarine suppressed EPSCs about equally in the glycinergic neurons from M_2_^+/+^ mice (*n* = 5 from five mice, 53.0 ± 6.7% of control) and from M_2_^−/−^ mice (*n* = 4 from three mice, 49.4 ± 4.8% of control; *p* = 0.68; Figure 10D).

**Figure 11:**
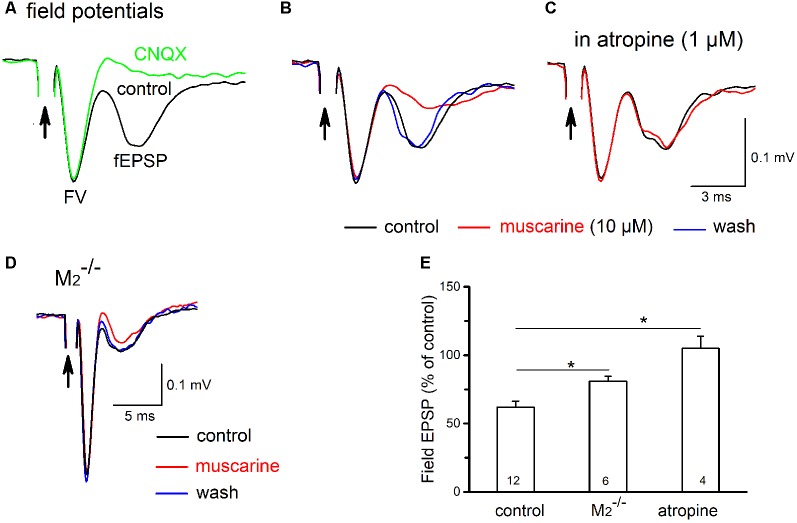
Muscarine reduces field excitatory postsynaptic potentials (fEPSPs) in the preBötC. **(A)** Electrical stimulation (100 μs, 100 μA; indicated by arrow) of the adjacent reticular formation evoked fEPSPs in the preBötC, consisting of an early axonal component (fiber volley, FV) and a late synaptic component (fEPSP). All recordings were performed in the presence of picrotoxin and strychnine to block fast inhibitory synaptic inputs. The late component was sensitive to AMPA receptor blockade by CNQX (20 μM) as indicated by comparing the two superimposed traces. **(B)** Muscarine (10 μM) produced a reversible attenuation of the fEPSP (traces in control, muscarine and during wash superimposed). In the same recording session, we repeated muscarine application in the presence of atropine (1 μM) and under these conditions muscarine failed to affect fEPSP amplitude **(C)**. **(D)** Superimposed traces recorded from the preBötC of an M_2_^−/−^ mouse illustrate attenuation of muscarine fEPSPs suppression. Arrows in **(A–D)** indicate truncated stimulating artifacts. Each trace is an average of 6–10 consecutive recordings. **(E)** Histogram summarizes muscarine-induced changes in peak fEPSP amplitude, expressed as a percentage of control values. The partial involvement of M_2_R is indicated by the reduced muscarinic effect in M_2_^−/−^ mice which were intermediate between atropine (no effect) and control. Numbers in columns represent sample number in each group (*p < 0.05).

In both types of preBötC neurons, the uniform suppression of evoked EPSCs by muscarine was accompanied by an appreciable shift of holding current. The majority of neurons exhibited an outward current shift during muscarine (9 out of 11 glycinergic neurons, 13.4 ± 4.3 pA; Figures 10E,F; 11 out of 17 non-glycinergic neurons, 27.5 ± 6.1 pA; Figure 10F). The remaining neurons showed either an inward current shift (two glycinergic and five non-glycinergic neurons) or no change (one non-glycinergic neuron). This response profile was profoundly changed when M_2_Rs were pharmacologically blocked or genetically disrupted. Under this condition, muscarine produced predominantly an inward current shift in both types of neurons (10 out of 12 glycinergic neurons, −13.5 ± 3.7 pA; Figures 10E,F; six out of nine non-glycinergic neurons, −10.3 ± 2.6 pA; Figure 10F), with no change in few neurons (two glycinergic and three non-glycinergic neurons).

### Muscarinic Suppression of Field EPSPs in the PreBötC

Finally, we examined whether muscarinic receptor activation would redistribute the strength of excitatory input between glycinergic and non-glycinergic preBötC neurons. For this purpose, we recorded pharmacologically isolated fEPSPs in the preBötC. Upon nearby electrical stimulation, EPSPs from different types of synaptically excited neurons should contribute to the recorded fEPSPs, but we do not know their relative weights. For the sake of simplicity, we neglect purely GABAergic neurons and assume that we are dealing here with a field potential largely generated by M_2_R-sensitive EPSCs in non-glycinergic neurons and by M_1_R-sensitive EPSCs in glycinergic neurons. The fEPSPs were relatively small in amplitude but easily identifiable (0.138 ± 0.077 mV, *n* = 12), and they were predominantly mediated by glutamatergic AMPA receptors, as suggested by their sensitivity to the specific AMPA receptor antagonist CNQX (30 μM, *n* = 4; Figure 11A). Muscarine (10 μM) reduced the peak amplitude of fEPSPs from 0.138 ± 0.077 mV to 0.085 ± 0.008 mV (*n* = 12, *p* = 0.00002, paired *t*-test; i.e., a decrease of 38.0 ± 4.4%; Figures 11B,E). This attenuation of fEPSPs by muscarine was blocked by the non-specific mAChR antagonist atropine (1 μM, *n* = 4, 105.1 ± 9.1% of control; Figures 11C,E). Because muscarinic inhibition of the excitatory drive onto the two cell types displays such strikingly differential receptor-subtype sensitivity (Figure 10), we reasoned that a comparison of muscarinic reduction of fEPSPs in the preBötC from wild type vs. M_2_R-deficient mice would be a way to predict whether glutamatergic input onto one neuronal player would be preferentially affected. We found that the lack of M_2_Rs significantly attenuated the inhibitory effect of muscarine on fEPSPs (Figures 11D,E). Muscarine caused a 19.0 ± 3.6% reduction of fEPSPs in M_2_^−/−^ mice (*n* = 6), suggesting that M_2_ receptors account for half (19.0 vs. 38.0%) of the muscarinic inhibition of preBötC excitatory neurotransmission. Based on our whole-cell data, muscarinic inhibition of fEPSPs in M_2_R-deficient preBötC should solely arise from the reduced excitatory input onto glycinergic neurons. Since the muscarinic inhibition in M_2_R^−/−^ preBötC represents about half of the overall fEPSP suppression in the wild-type counterpart, it seems plausible to assume that muscarinic receptor activation should equally dampen the synaptic excitation of glycinergic and non-glycinergic neurons.

## Discussion

In the present study, we characterized the electrophysiological properties and morphological features of putative glycinergic neurons in the preBötC in slices from young (postnatal day 6–13) mice. Our detailed morphometric analysis of reconstructed preBötC glycinergic neurons addresses existing gaps regarding the morphology and axonal arborizations of this functionally important neuronal population. We identified a significant population (32%) of glycinergic neurons which have axon collaterals that extend into ipsilateral premotor/motor areas and/or the contralateral preBötC. Interestingly, these neurons have more primary dendrites and a more compact dendritic tree than glycinergic neurons that have axon/collaterals that remain within their own dendritic fields in the preBötC. Spontaneous AP firing was modulated by mAChR activation in the preBötC glycinergic neurons, with M_2_R-mediated reduction of firing being more prevalent than M_1_R-mediated excitation. Furthermore, we provide evidence that muscarinic receptor activation dampens both inhibitory and excitatory synaptic inputs onto glycinergic neurons, unexpectedly involving mAChRs of the M_1_ family.

### The Activity of PreBötC Glycinergic Neurons *in vitro*

The preBötC contains heterogeneous populations of glutamatergic excitatory and GABAergic/glycinergic inhibitory neurons (Winter et al., 2009; Koizumi et al., 2013). Glycinergic neurons are estimated to constitute about 20–55% of all preBötC neurons, with less than half of them being coupled to inspiration (Manzke et al., 2010; Morgado-Valle et al., 2010). A substantial subpopulation of inhibitory neurons expresses both glycine and GABA (Koizumi et al., 2013; Rahman et al., 2013; Hirrlinger et al., 2019). The majority of preBötC glycinergic neurons recorded in this study in mouse brainstem slices fired spontaneously with regular repetitive APs under our experimental conditions, where excitatory synaptic transmission was left intact and GABAergic inhibition was blocked. Only a small fraction (6%) of neurons fired APs in rhythmic “bursts.” Rhythmic inspiratory neurons in the preBötC are usually identified by their firing in phase with inspiratory bursts recorded from XIIn, and pacemaker neurons are a subset of inspiratory neurons which have intrinsic voltage-dependent bursting properties (Smith et al., 1991; Del Negro et al., 2002; Peña et al., 2004; Koizumi et al., 2013). Despite the unidentified respiratory relationship of our recorded glycinergic neurons (without XIIn monitoring), a few neurons did fire in a rhythmic “burst-like” pattern in our study. We found two distinct modes of “bursting,” with some neurons firing brief “bursts” consisting of a few APs superimposed on a small envelope of membrane depolarization (Figure 7B), and others that showed intensive discharges riding on top of a strong and prolonged membrane potential depolarization (Figure 7A), which may resemble inspiratory neurons with or without burst properties, respectively (Tryba et al., 2003; Pace et al., 2007).

Using calcium imaging, Winter et al. (2009) reported that about 20% of EGFP-labeled glycinergic neurons show inspiratory calcium signals and these make up half of the inspiratory neurons in the preBötC. Whole-cell recordings from rhythmic slices bathed in elevated potassium (8–9 mM) showed that about 40% of EGFP-labeled glycinergic neurons displayed respiratory-modulated discharges, while the remainder exhibited either silent or irregular firing patterns (Morgado-Valle et al., 2010) or a “tonic” firing pattern (Winter et al., 2009). It is well-known that activity in preBötC depends on its relative location in the rhythmic slices, slice thickness and importantly, on the potassium concentration in the superfusate (Tryba et al., 2003; Ruangkittisakul et al., 2006, 2011; Bacak et al., 2016). The lower incidence of rhythmic neurons in our hands could be attributable to the relatively thin slices (300–350 μm thick) and an external solution with a physiological potassium concentration (3 mM). Even in precisely calibrated slices, with a center-located preBötC, the rhythm observed in thicker slices declined steadily over time when the slices were bathed in solution with a physiological potassium concentration (Ruangkittisakul et al., 2006, 2011).

### Morphological Features of PreBötC Glycinergic Neurons

In contrast to the extensively explored rhythmogenic properties of the preBötC, our understanding of correlations between electrophysiological and morphological properties of neurotransmitter-identified neurons in this brain region is still incomplete (Koizumi et al., 2013). Glycinergic neurons were distributed widely throughout the preBötC region, and they varied widely in size, as shown both with membrane capacitance measurements and by morphological analysis of reconstructed somata. Since our targeted whole-cell recordings were limited to EGFP-labeled neurons located in the uppermost layer of the slice (up to 100 μm deep), our morphometric analysis was confined to a population of cells whose axons and dendrites might have been cut off during the slicing procedure. A few primary dendrites (mostly spine-free) reached about 500 μm into the ventral respiratory column. Axons started either directly from the soma or at a short distance from primary dendrites, and axonal collaterals remained mainly ipsilaterally within dendritic fields or ran towards premotor/motor areas (e.g., XIIn pre- and motor-neurons or bulbospinal rostral ventral respiratory group circuits). Such an axonal organization fits well with their proposed roles in local phasic inhibition and feed-forward inhibition along the inspiratory drive transmission pathway (Koizumi et al., 2013). Consistent with the minor commissural projections of inhibitory neurons found by others (Koizumi et al., 2013), we only infrequently observed axonal projections to the contralateral preBötC, accompanied by extensive collaterals in the ipsilateral premotor area (Figure 4A). This suggests a possible synchronous modulation by glycinergic neurons of postsynaptic targets located on both sides of the brainstem respiratory area. We also found that some glycinergic neurons have axonal projections to the Nucleus ambiguus, suggesting that they might have cardiovagal effects (Hou et al., 2009).

In many previous studies, inspiratory-modulated neurons in the preBötC were assumed to be excitatory in nature because they lacked neurotransmitter identification (Smith et al., 1991; Del Negro et al., 2002; Picardo et al., 2013; Zavala-Tecuapetla et al., 2014). Recently, the structural-functional properties of neurotransmitter-identified inspiratory neurons have been characterized (St-John et al., 2009; Koizumi et al., 2013). Interestingly, the dendritic fields of preBötC inhibitory inspiratory neurons are more spatially compact than those of neighboring excitatory neurons (Koizumi et al., 2013). In our study we compared the dendritic arborizations of inhibitory glycinergic neurons, sub-grouped according to the axonal projection patterns, and our analysis revealed that preBötC glycinergic neurons with axonal collaterals extending away from dendritic fields have more primary dendrites and more compact dendritic fields compared to those neurons with axon/collaterals located mostly within their own dendritic fields. This may indicate a difference in information integration areas between the two groups of glycinergic neurons.

### Muscarinic Modulation of PreBötC Glycinergic Neurons

Acetylcholine plays an important role in respiration, both peripherally and centrally, ranging from respiratory muscle contraction and chemosensation, to control of neuronal excitability within the rhythm-generating network (Bellingham and Ireland, 2002). The principal cholinergic projection system in the brainstem originates in the pedunculopontine tegmental nucleus and the laterodorsal tegmental nucleus, with a contribution of local cholinergic neurons found in the medullary reticular formation and near the ventral medullary surface (reviewed in Shao and Feldman, 2009). Both nicotinic and mAChRs are implicated in facilitating respiratory activity by acting on brainstem respiratory-related circuits including the preBötC and hypoglossal motoneurons (Bellingham and Berger, 1996; Shao and Feldman, 2000, 2005, 2009; Pagnotta et al., 2005). At the network level, muscarinic receptor activation produces diverse effects, such as increased sighs and reduced eupneic activity (Shao and Feldman, 2000, 2005; Tryba et al., 2008; Koch et al., 2013). Among regular spiking glycinergic neurons, we found that the majority of glycinergic neurons displayed either a sustained decrease in firing rate or an initial decrease followed by an increase in firing during muscarinic activation; only 17% increased their firing rate. These inhibitory and excitatory effects involved gallamine-sensitive M_2_Rs and pirenzepine-sensitive M_1_Rs, respectively. Taking into account that M_2_ receptors are the most prevalent among muscarinic receptor subtypes in the brainstem including the preBötC (Levey, 1993; Lai et al., 2001), it is not surprising to find an M_2_ receptor-mediated suppression of firing in the majority of regular spiking glycinergic neurons. Interestingly, in our limited sample of “burst-like” firing glycinergic neurons in the preBötC, we observed a muscarinic enhancement of rhythmicity accompanied by membrane depolarization, which is consistent with the M_3_R-mediated postsynaptic excitation seen in neurotransmitter-undefined inspiratory neurons (Shao and Feldman, 2000). In one glycinergic neuron, muscarine led to a depolarization block during burst firing, which might involve a calcium-activated non-specific cation current (Picardo et al., 2013). PreBötC bursting pacemaker neurons in the respiratory network are heterogeneous (Thoby-Brisson and Ramirez, 2001; Peña et al., 2004), and are differentially modulated by a variety of neuromodulators like norepinephrine and 5-HT (Peña and Ramirez, 2002; Viemari and Ramirez, 2006). A larger sample of the rare rhythmic bursters among the glycinergic neurons would be needed to define the mechanism behind their muscarinic modulation and to determine whether muscarine would produce similar effects on the few glycinergic pacemaker neurons identified by Morgado-Valle et al. (2010) in the neonatal preBötC slice in high extracellular K^+^ concentration (9 mM).

The mixed effects of muscarinic activation on preBötC neuronal firing could be the result of muscarinic actions on intrinsic firing properties and/or the result of modulation of synaptic inputs. Since glutamatergic synaptic transmission during recordings of spontaneous firing was left intact, we cannot exclude the possibility that the muscarinic effects on spontaneous firing in glycinergic neurons might have involved a network effect through altered excitatory neurotransmission. Indeed, we found that muscarine suppressed excitatory synaptic inputs onto preBötC neurons, similar to what has been described for evoked EPSCs in hypoglossal motoneurons (Bellingham and Berger, 1996). Interestingly, genetic disruption of M_2_Rs or application of an M_2_R antagonist in wild type preBötC did not affect the muscarinic suppression of EPSCs in glycinergic neurons, but abolished that in non-glycinergic neurons. The opposite effect was seen with an M_1_R antagonist, namely a block of muscarinic depression in glycinergic neurons and little effect on EPSCs in non-glycinergic neurons. Together with the field EPSP recordings, in which the lack of M_2_Rs attenuated muscarinic inhibition by half, these data suggest the existence of an equally effective, but mechanistically different muscarinic inhibition of excitatory synaptic inputs for different types of preBötC neurons.

Glycinergic neurons themselves are under strong inhibitory synaptic control (Winter et al., 2009), and we found that the inhibitory synapses onto these cells are subject to a strong PPD. Muscarinic AChR activation down-regulated evoked IPSCs and turned the characteristic PPD into PPF. This was mainly due to a dramatic reduction in the amplitude of the first evoked IPSC. Similar findings have been described for muscarinic modulation of hippocampal GABAergic synapses (Seeger et al., 2004; González et al., 2014), an effect that was attributed to a presynaptic site. While this action of muscarine on inhibitory synaptic transmission suggests a presynaptic mechanism, we found that both the amplitude and frequency of spontaneous IPSCs is reduced by muscarine in most glycinergic neurons (unpublished observations), suggesting an involvement of both pre- and postsynaptic modulatory sites.

**Figure 12:**
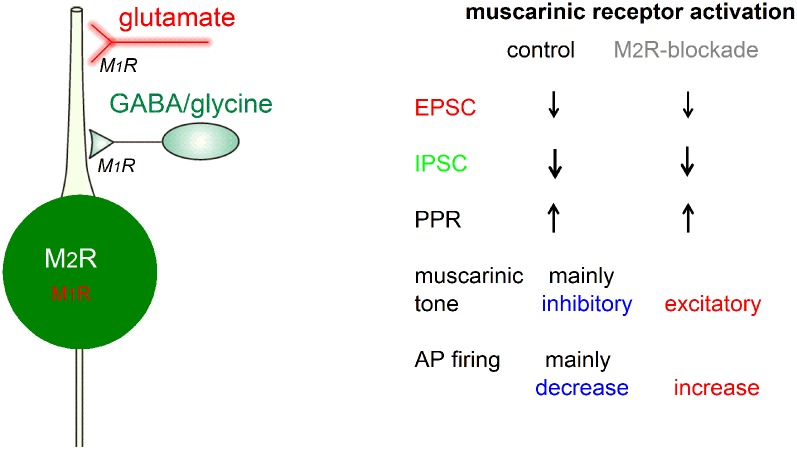
Graphic summary of muscarinic effects on preBötC glycinergic neurons. For explanation see text. Abbreviations: AP, action potential; EPSC, excitatory postsynaptic current; IPSC, inhibitory postsynaptic current; M_1_R or M_2_R, muscarinic acetylcholine receptor type 1 or 2; PPR, paired-pulse ratio.

A graphic summary of our main findings regarding the muscarinic modulation of preBötC glycinergic neurons is depicted in Figure 12. Generally, mAChRs reduce synaptic drive onto these neurons mainly through presynaptic M_1_-like receptors, with a stronger effect on inhibitory than on excitatory inputs. On the postsynaptic site, M_2_R-mediated inhibition of spontaneous firing prevailed over M_1_R-mediated excitation in the vast majority of glycinergic neurons.

### Functional Implications of Muscarinic Modulation of Inhibitory Networks Within the PreBötC

The muscarine-induced switch from PPD to PPF of evoked IPSCs/EPSCs in glycinergic neurons alters the way in which these neurons respond to repeated synaptic stimulation, turning them from a low-pass synaptic filter (showing PPD) to a band-pass filter (showing PPF). Together with our observation that IPSCs are down-regulated more than EPSCs, this suggests that mAChR activation strengthens the overall synaptic excitation of these neurons, thereby increasing synaptic inhibitory output. Our laboratory has shown previously that activation of presynaptic M_2_Rs depressed (GABAergic) IPSCs more potently than EPSCs in hippocampal CA1 pyramidal cells, similar to the findings in the present study, and that the lack of this disinhibitory mechanism underlies the impaired synaptic plasticity seen in M_2_^−/−^ mice (Seeger et al., 2004).

The overall effects of muscarine on the output of glycinergic neurons depends on a complex combination of actions, including the regulation of the balance between synaptic excitation and inhibition, and postsynaptic actions related to control of intrinsic membrane properties through modulation of voltage-gated ion channels regulating resting membrane potential and excitability. Thus, we cannot make firm predictions about the global impact of mAChR activation in the intact respiratory network. What our study demonstrates are apparently opposing effects of muscarine, augmenting firing in a limited number of “burst-like” preBötC glycinergic neurons, while dampening firing in regular spiking glycinergic neurons. This may represent a means of physiological cholinergic input to enhance the “signal-to-noise ratio” of glycinergic inhibition. Overstimulation of the cholinergic nervous system—as occurs after intoxication by pesticides or nerve agents, which inhibit acetylcholinesterase—will then progressively disorganize the normal respiratory patterns and eventually cause respiratory arrest.

## Data Availability Statement

The datasets generated for this study are available on request to the corresponding author.

## Ethics Statement

The animal study was reviewed and approved by Government of Middle Franconia, Bavaria, Germany.

## Author Contributions

FZ conducted voltage-clamp and field potential recordings and performed data analysis. BN-B conducted current-clamp recordings and performed morphological analysis. EE and JB participated in the initial study. CA and FZ designed and supervised the project. CA wrote the article, with participation from BN-B, JB and FZ.

## Conflict of Interest

The authors declare that the research was conducted in the absence of any commercial or financial relationships that could be construed as a potential conflict of interest.

## Abbreviations

aCSF: artificial cerebrospinal fluid
AP: action potential
EGFP: enhanced green fluorescent protein
EPSC: excitatory postsynaptic current
fEPSP: field excitatory postsynaptic potential
FV: fiber volley
GlyT_2_: glycine transporter type 2
IPSC: inhibitory postsynaptic current
mAChR: muscarinic acetylcholine receptor
PPD: paired-pulse depression
PPF: paired-pulse facilitation
PPR: paired-pulse ratio
preBötC: pre-Bötzinger complex.
